# Comorbid Chronic Pain and Post-traumatic Stress Disorder: Current Rates and Psychiatric Comorbidities Among U.S. Military Veterans

**DOI:** 10.1093/milmed/usae313

**Published:** 2024-06-13

**Authors:** Katherine Hadlandsmyth, Caywin Zhuang, Mary A Driscoll, Brian C Lund

**Affiliations:** VA Office of Rural Health (ORH), Veterans Rural Health Resource Center-Iowa City, Iowa City VA Health Care System, Iowa City, IA 52246, USA; Center for Access & Delivery Research and Evaluation (CADRE), Iowa City VA Health Care System, Iowa City, IA 52246, USA; Department of Anesthesia, University of Iowa Carver College of Medicine, Iowa City, IA 52242, USA; Department of Anesthesia, University of Iowa Carver College of Medicine, Iowa City, IA 52242, USA; Pain Research, Informatics, Multimorbidities, and Education (PRIME) Center, VA Connecticut Healthcare System, West Haven, CT 06516, USA; Department of Psychiatry, Yale School of Medicine, New Haven, CT 06511, USA; VA Office of Rural Health (ORH), Veterans Rural Health Resource Center-Iowa City, Iowa City VA Health Care System, Iowa City, IA 52246, USA; Center for Access & Delivery Research and Evaluation (CADRE), Iowa City VA Health Care System, Iowa City, IA 52246, USA; Department of Epidemiology, University of Iowa College of Public Health, Iowa City, IA 52242, USA

## Abstract

**Introduction:**

This study reports rates of comorbid chronic pain and post-traumatic stress disorder (PTSD) among U.S. military veterans and rates of psychiatric comorbidities among those with both chronic pain and PTSD.

**Materials and Methods:**

This study utilized National Veterans Affairs (VA) administrative data to identify all veterans treated for chronic pain or PTSD in 2023. Multivariable logistic regression models determined the likelihood of each psychiatric comorbidity for those with chronic pain and PTSD relative to those with chronic pain only and separately to those with PTSD only, after adjusting for demographic variables and all other psychiatric comorbidities.

**Results:**

Of the 5,846,453 service users of the VA in 2023, a total of 2,091,391 (35.8%) met the criteria for chronic pain and 850,191 (14.5%) met the criteria for PTSD. Furthermore, 21.6% of those with chronic pain also had PTSD and over half (53.2%) of those with PTSD also met the criteria for chronic pain (*n* = 452,113). Veterans with chronic pain and PTSD were significantly more likely to be women, Black or African American, Hispanic or Latina, and urban dwelling. Veterans with chronic pain and PTSD had significantly higher rates of all selected comorbidities relative to veterans with chronic pain only.

**Conclusions:**

Patients with comorbid chronic pain and PTSD may benefit from tailored treatments to address the additive impact of these conditions.

## INTRODUCTION

Per the mutual maintenance model, post-traumatic stress disorder (PTSD) can maintain and magnify the impairing impact of chronic pain.^1–6^ Similarly, chronic pain can worsen the impact of PTSD.^1–6^ Previous studies examining the impact of these comorbidities on one another have frequently compared those with chronic pain and PTSD (CP + PTSD) to either those with chronic pain only or those with PTSD only. As such, it is not clear whether those with CP + PTSD are truly unique or whether they are more similar to either those with chronic pain or those with PTSD alone.

One important subset of patients, U.S. military veterans, are at heightened risk for developing both CP + PTSD, making them a vulnerable group for developing this comorbidity.^7,8^ The objective of the current study is to report current rates of chronic pain, PTSD, and CP + PTSD in U.S. military veterans in 2023. To characterize the subset of patients with both CP + PTSD, they are then compared to both those with chronic pain alone and those with PTSD alone on demographic variables and other psychiatric comorbidities.

## METHODS

### Data Sources

This study used National Veterans Affairs (VA) administrative data from the corporate data warehouse to identify all VA service users in 2023 (i.e., those with at least two outpatient visits in 2023). Chronic pain was defined using previously established methods, as two or more ICD-10 diagnoses indicating chronic pain; at least one diagnostic code indicating chronic pain and two or more numeric rating scales ≥4; or long-term opioid use (>90 days).^9–15^ PTSD was also defined using previously defined methods, requiring at least one inpatient ICD-10 code of F43.1x in 2023, or at least two outpatient codes of F43.1x: one in 2023 and a second in the prior 730 days.^10,16,17^ Demographic data were extracted from administrative data files. Psychiatric comorbidities were determined by the presence of an inpatient hospitalization or outpatient encounter in 2023 associated with an ICD-10 code indicating the comorbidity.^10^ This study was submitted to the local institutional review board for a human subjects research determination, was determined to not constitute human subjects research, and was thus exempt.

### Analyses

Frequencies of demographic characteristics were reported for those with both CP + PTSD, those with chronic pain only, and those with PTSD only. Univariate logistic regression models determined differences between those with CP + PTSD relative to those with chronic pain only and separately to those with PTSD only on demographic variables. Multivariable logistic regression models were used to determine the likelihood of each psychiatric comorbidity for those with CP + PTSD relative to those with chronic pain only and separately to those with PTSD only, after adjusting for all demographic variables and all other psychiatric comorbidities. To account for multiple comparisons, a significance threshold of α = .001 was used for bivariate comparisons and correspondingly, a 99.9% CI was reported for logistic regression odds ratio estimates.

## RESULTS

Among the 5,846,453 service users of the VA in 2023, a total of 2,091,391 (35.8%) met the criteria for chronic pain and 850,191 (14.5%) met the criteria for PTSD. A total of 452,113 met the criteria for both CP + PTSD, meaning that 21.6% of those with chronic pain also had PTSD and over half (53.2%) of those with PTSD also met the criteria for chronic pain. The average age of those with CP + PTSD (55 years) fell in between the average age of those with chronic pain (62 years) and those with PTSD (51 years). Veterans with CP + PTSD were significantly more likely to be women, Black or African American, Hispanic or Latina, and urban dwelling relative to those with chronic pain only or PTSD only (**Table 1**).

**Table 1.** Demographics among those with chronic pain, PTSD, or both (*N* = 2,489,469).

Variable	CP + PTSD (*n* = 452,113), *n* (%) or *M* (SD)	Chronic pain^a^ (*n* = 1,639,278), *n* (%) or *M* (SD)	PTSD^a^ (*n* = 398,078), *n* (%) or *M* (SD)
Age	55.1 (15.3)	62.4 (15.4)	51.1 (16.3)
Sex			
Female	84,937 (18.8)	191,948 (11.7)	63,555 (16.0)
Male	367,176 (81.2)	1,447,330 (88.3)	334,523 (84.0)
Racial identification			
Black or African American	115,580 (25.6)	360,079 (22.0)	85,628 (21.5)
Asian American	7,478 (1.7)	24,705 (1.5)	7,540 (1.9)
American Indian or Alaskan Native	6,653 (1.5)	16,530 (1.0)	5,806 (1.5)
Native Hawaiian or Pacific Islander	6,132 (1.4)	17,894 (1.1)	5,218 (1.3)
White American	287,190 (63.5)	1,111,655 (67.8)	263,441 (66.2)
Unknown	29,080 (6.4)	108,415 (6.6)	30,455 (7.6)
Hispanic or Latino ethnicity	48,352 (10.7)	129,656 (7.9)	41,741 (10.5)
Residence			
Rural	135,269 (29.9)	510,657 (31.2)	122,981 (30.1)
Urban	316,844 (70.1)	1,128,621 (68.9)	275,097 (69.1)

a Across all demographic variables, those with chronic pain only or PTSD only differed significantly from those with CP + PTSD at the *P* < .001 level.

Veterans with CP + PTSD had significantly higher rates of all selected comorbidities relative to veterans with CP only, with the largest differences observed for depressive disorders (adjusted odds ratio [aOR] = 3.43, 99.9% CI, 3.39-3.47) and panic disorder (aOR = 2.16, 99.9% CI, 2.09-2.25) (**Table 2**). Statistical differences among veterans with CP + PTSD were also observed for most comorbidities relative to those with PTSD only, but the effect sizes were generally much smaller. For example, for generalized anxiety disorder, the aOR for CP + PTSD versus CP only was 1.26 (95% CI, 1.23-1.28) compared to 1.07 (95% CI, 1.04-1.09) for CP + PTSD versus PTSD only. An exception to this pattern was sleep disorders, where aOR estimates were higher for CP + PTSD versus PTSD only (aOR = 2.03, 99.9% CI, 2.00-2.06) than for CP + PTSD versus CP only (aOR = 1.84, 99.9% CI, 1.81-1.86).

**Table 2.** Prevalence of selected comorbidities among veterans with chronic pain and PTSD relative to those with chronic pain only or PTSD only.

	CP and PTSD	Chronic pain only		PTSD only	
Comorbidity	Frequency, *n* (%)	Frequency, *n* (%)	aOR (99.9% CI)^a^	Frequency, *n* (%)	aOR (99.9% CI)^a^
Depressive disorder	293,147 (64.8)	453,182 (27.6)	3.43 (3.39-3.47)	229,993 (57.8)	1.27 (1.25-1.29)
Generalized anxiety disorder	57,485 (12.7)	87,756 (5.4)	1.26 (1.23-1.28)	45,145 (11.3)	1.07 (1.04-1.09)
Panic disorder	19,290 (4.3)	18,609 (1.1)	2.16 (2.09-2.25)	15,178 (3.8)	1.06 (1.02-1.10)
Obsessive compulsive disorder	4,793 (1.1)	6,190 (0.4)	1.63 (1.53-1.75)	3,667 (0.9)	1.06 (0.98-1.14)
Alcohol use disorder	83,461 (18.5)	123,129 (7.5)	1.84 (1.81-1.87)	77,395 (19.4)	0.92 (0.90-0.94)
Opioid use disorder	14,814 (3.3)	22,042 (1.3)	1.41 (1.36-1.47)	10,815 (2.7)	1.23 (1.17-1.28)
Other substance use disorder	48,324 (10.7)	63,129 (3.9)	1.54 (1.50-1.58)	41,046 (10.3)	1.07 (1.04-1.10)
Sleep disorder	268,877 (59.5)	637,780 (38.9)	1.84 (1.81-1.86)	167,170 (42.0)	2.03 (2.00-2.06)

Abbreviation: aOR, adjusted odds ratio.

a aORs are expressed for patients with chronic pain and PTSD, relative to patients with chronic pain only, and separately to patients with PTSD only. Separate multivariable logistic regression models were created for each comorbidity individually as the dependent variable and adjusted for all demographic variables and all other comorbidities.

## DISCUSSION

The current findings indicate high rates of comorbidity of CP + PTSD among VA-served veterans: about one in five of those with chronic pain met the criteria for PTSD. This is higher than rates of PTSD previously reported in civilian pain populations of 10-12%.^18^ Furthermore, over half of those with PTSD met the criteria for chronic pain, which is consistent with prior reports ranging from 25% to 80%.^19^ Additionally, rates of women and African Americans were higher among those with CP + PTSD relative to either condition alone.

Current findings also speak to greater complexity among patients with CP + PTSD, via an additive effect of risk for additional psychiatric comorbidities. Sleep disorder and depressive disorders, in particular, were strikingly higher among those with CP + PTSD relative to those with either condition alone. This is consistent with prior work which has demonstrated that poor sleep quality can exacerbate the magnifying impact of PTSD symptoms on chronic pain.^20^ The odds of opioid use disorder were also significantly heightened, such that those with CP + PTSD were 1.4 times as likely to have opioid use disorder relative to those with chronic pain alone and 1.2 times more likely relative to those with PTSD alone.

This work has some limitations. Findings from a VA population may not fully generalize to other populations. Further, while administrative data can illustrate trends at a population level, these data do not speak to the needs of the individual patient. As such, subsequent work is needed to determine how to best meet the needs of veterans with CP + PTSD.

In conclusion, these findings suggest a high prevalence of overlapping CP + PTSD among VA-served U.S. military veterans and indicate greater psychiatric complexity in this population. Patients with both CP + PTSD may benefit from tailored treatments to address the additive impact of these conditions. Additionally, given the over-representation of women and African American/Black Veterans in the CP + PTSD group, efforts to better understand the unique risks for these populations are necessary if we are to adequately address the highlighted disparities.

## Data Availability

These data are not available on request to authors, and they belong to the Veterans Health Administration who can grant access.

## INSTITUTIONAL CLEARANCE

Institutional clearance approved.
